# Diabetes Complications at Presentation and One Year by Glycated Haemoglobin at Diagnosis in a Multiethnic and Diverse Socioeconomic Population: Results from the South London Diabetes Study

**DOI:** 10.1155/2015/587673

**Published:** 2015-05-18

**Authors:** Mohsin Azam, Lindsey Marwood, Khalida Ismail, Tyrrell Evans, Sobha Sivaprasad, Kirsty Winkley, Stephanie Anne Amiel

**Affiliations:** ^1^Division of Diabetes and Nutritional Sciences, King's College London, Diabetes Research Group, Weston Education Centre, 10 Cutcombe Road, London SE5 9RJ, UK; ^2^Department of Psychological Medicine, King's College London, Weston Education Centre, 10 Cutcombe Road, London SE5 9RJ, UK; ^3^Paxton Green Group Practice, 1 Alleyn Park, London SE21 8AU, UK; ^4^Department of Ophthalmology, King's College Hospital NHS Foundation Trust, Denmark Hill, London SE5 9PJ, UK

## Abstract

*Background*. WHO's recommendation of HbA_1c_ ≥ 48 mmol/mol (6.5%) as diagnostic for type 2 diabetes mellitus (T2DM) was adopted by three UK London boroughs in May 2012. The South London Diabetes (SOUL-D) study has recruited people with newly diagnosed T2DM since 2008. We compared participants diagnosed before May 2012 with HbA_1c_ < 48 mmol/mol to those with diagnostic HbA_1c_ ≥ 48 mmol/mol. *Methods*. A prospective cohort study of newly diagnosed T2DM participants from 96 primary care practices, comparing demographic and biomedical variables between those with diagnostic HbA_1c_ < 48 mmol/mol or HbA_1c_ ≥ 48 mmol/mol at recruitment and after one year. *Results*. Of 1488 participants, 22.8% had diagnostic HbA_1c_ < 48 mmol/mol. They were older and more likely to be white (*p* < 0.05). At recruitment and one year, there were no between-group differences in the prevalence of diabetic complications, except that those diagnosed with HbA_1c_ < 48 mmol/mol had more sensory neuropathy at recruitment (*p* = 0.039) and, at one year, had new myocardial infarction (*p* = 0.012) but less microalbuminuria (*p* = 0.012). *Conclusions*. Use of HbA_1c_ ≥ 48 mmol/mol as the sole T2DM diagnostic criterion may miss almost a quarter of those previously diagnosed in South London yet HbA_1c_ < 48 mmol/mol may not exclude clinically important diabetes.

## 1. Introduction

The use of plasma glucose, measured after fasting and a standardised oral glucose load, for the diagnosis of type 2 diabetes (T2DM) has long been considered inconvenient [1]. Glycated haemoglobin (HbA_1c_), formed in a nonenzymatic reaction between glucose and haemoglobin and reflecting a 2-3-month average of plasma glucose concentrations in a single random sample [2], was considered as an alternative diagnostic tool for T2DM by an International Expert Committee in 2009 [1]. It has both advantages and disadvantages, [2] but in 2010 and 2011, respectively, both the American Diabetes Association (ADA) [3] and World Health Organisation (WHO) [4] proposed that HbA_1c_ ≥ 48 mmol/mol (6.5%) is diagnostic of T2DM, based on successive reproducible data from a number of publications linking HbA_1c_ concentrations and diabetes specific complications, notably retinopathy [5–8].

Prior to this, people in South London, in the United Kingdom [9], were diagnosed with diabetes by plasma glucose measurements, with or without formal glucose tolerance tests, although HbA_1c_ was measured at diagnosis for assessment and monitoring purposes. The authorities providing healthcare to a large area of South London did not adopt WHO's recommendations of HbA_1c_ ≥ 48 mmol/mol (6.5%) as the diagnostic tool for T2DM until May 2012 [10]. Four years earlier, in May 2008, the South London Diabetes Study (SOUL-D) had begun recruiting adults with new-onset diabetes from 96 primary care practices in the London boroughs of Lambeth, Southwark, and Lewisham into an observational cohort study [11]. The majority were diagnosed prior to May 2012 using plasma glucose values. SOUL-D created an opportunity to examine how well HbA_1c_ would perform compared to previously used methods for diagnosing diabetes in this multiethnic, high-risk population.

The aims of this study were to examine the proportion of participants in the SOUL-D cohort that were diagnosed with HbA_1c_ < 48 mmol/mol (6.5%) before the new guidelines were introduced; determine whether these participants were significantly different from participants with higher HbA_1c_ at diagnosis in their demography and diabetes complications; and investigate the criteria used to diagnose diabetes in patients with HbA_1c_ < 48 mmol/mol (6.5%).

## 2. Patients and Methods

### 2.1. Subjects

One thousand seven hundred and fifteen people with newly diagnosed T2DM diagnosed before May 2012 were recruited from 96 (70% eligible) consenting primary care practices in the London boroughs of Lambeth, Southwark, and Lewisham, which serve a diverse population of almost one million residents. The ethnic origin of the boroughs' population is 66.6% white, 20% black, and 13.4% South Asian [9]. Eligible participants were aged 18–75 years and had been diagnosed with T2DM within the last 6 months. Exclusion criteria included being a temporary resident, people diagnosed with diabetes other than T2DM, known severe mental illness (dementia, bipolar disorder, substance dependence, and personality disorder), severe advanced diabetes complications (e.g., being registered blind, requiring dialysis, or above knee amputation), and those not fluent in English (estimated at 7% [9]).

### 2.2. Methods

This was a prospective cohort study comparing 2 participant groups: those diagnosed with T2DM with HbA_1c_ < 48 mmol/mol (6.5%) and those diagnosed with HbA_1c_ ≥ 48 mmol/mol (6.5%). The protocol for recruitment of participants in SOUL-D has been published previously [11], but, in brief, potential participants were identified by 6 monthly practice database reviews and invited to participate if they had a new diagnosis of T2DM.

Consenting patients attended their primary care practice to meet a SOUL-D researcher with whom they completed a standardised interview including medical history, employment status, education history (years of education attended), physical examination, and blood test. This was completed within 6 months of diagnosis (recruitment data) and 12 months after recruitment (one-year data). Additional clinical data from the time of diagnosis (diagnostic data), including HbA_1c_, body mass index (BMI), blood pressure, urinary albumin : creatinine ratio (ACR), and lipid profile (low-density lipoprotein (LDL), total cholesterol, high-density lipoprotein (HDL), and triglycerides), were extracted from each participant's medical records to give preintervention parameters. Cross-sectional analysis was undertaken to compare diagnostic and recruitment clinical and sociodemographic variables between those participants whose diagnostic HbA_1c_ had been <48 mmol/mol and those with higher diagnostic values. Analysis of year 1 study follow-up data was also undertaken to see if early progression of diabetes (reflected by HbA_1c_ and treatment prescribed) varied between groups.

For cross-sectional baseline (diagnostic and recruitment) analysis, age, gender, and self-reported ethnicity (using the 2001 consensus classification [9]) were noted at recruitment. Date of diabetes diagnosis and HbA_1c_ at diagnosis were recorded from medical records. Macrovascular complications, myocardial infarction (MI) and stroke/cerebrovascular accident (CVA), were defined by history given at recruitment and confirmed by examination of medical records. BMI (kg/m^2^), blood pressure (mmHg), foot examination, and sensory neuropathy were determined at recruitment, the last using vibration perception threshold >25 volts measured by neurothesiometer (Scientific Laboratory Supplies, Wilford, Nottingham). Retinopathy status was obtained from the Diabetes Eye Complications Screening (DECS) service, the local community based eye photography service used in all three boroughs, using digital two-field retinal photographs and the English Retinopathy Minimum grading [12]. Fasting blood was taken for HbA_1c_ and lipid measurements. Microalbuminuria was defined on a single ACR measurement in the records of >3 *μ*g/mg. Medication status (presence or absence of oral hypoglycemic agents and insulin) at recruitment was recorded from patient history, confirmed from the participants' medical records.

One year follow-up assessment was made within a 3-month window of 12 months after recruitment. The data collection made at recruitment was repeated. If participants declined, data were obtained from their GP surgery (with permission) within a three-month window of their study appointment.

To determine the basis of diagnosis for each participant whose HbA_1c_ was <48 mmol/mol (6.5%) at diagnosis, additional specific permission was granted to access the clinical database of 43 GP surgeries, selected for having ≥5 participants meeting the above criteria. Patient records were examined for details of symptoms at diagnosis (polyuria, polydipsia, fatigue, blurred vision, and weight loss) and details of diagnostic testing (fasting plasma glucose (FPG), random plasma glucose (RPG), or oral glucose tolerance test (OGTT) glucose concentrations) were noted. The data were compared with the relevant WHO recommendations to assess the accuracy of the original diagnosis [6]. Case-by-case judgements on the diagnosis were made by 2 independent clinicians reviewing the data.

HbA_1c_ (%) at diagnosis was measured in one of 3 local laboratories according to IFCC methods (aligned with the DCCT) based on HPLC and then quantified during capillary electrophoresis or electron spray ionization mass spectrometry. The assay methods used were (1) the Trinity Biotech Ultra 2 boronate affinity chromatography (coefficient variations (CV%) 0.82%, 0.91%, and 0.46% for normal, intermediate, and high HbA_1c_ values based on 20 assays with the same run time), (2) the Trinity Biotech Premier Hb9210 analyser, also a boronate affinity chromatography-based high performance liquid chromatography system (CV% 1.62%, 1.59%, and 1.68% for low, medium, and high values, resp.), and (3) TOSOH G7 ion exchange with imprecision CV% less than or equal to 1.2%. For all three laboratories, the CV% was well below the recommended upper limit of 2% CV and there were no changes in the methodologies between 2008 and 2013. HbA_1c_ samples measured at recruitment and one year were all measured in the central laboratory (method 1). Percentage (%) values were converted to mmol/mol by subtracting 2.15 then multiplying by 10.929 [13]. Lipid profiles and ACRs were measured using Siemens ADVIA 2400. The PEG-enhanced immunoturbidimetric assay was used for urinary albumin and the Jaffe reaction for urinary creatinine.

*Statistical analysis* was performed using SPSS version 22 [14]. Data are presented as mean [standard deviation (SD)] where data were normally distributed, or median [interquartile range (IQR)] where data were skewed, which could not be corrected by log transforming the variables, or as a count (percentage) for categorical variables, all stratified by diagnostic HbA_1c_ status. Unadjusted statistical analyses, comparing participants diagnosed with HbA_1c_ < 48 mmol/mol (6.5%) to those with HbA_1c_ ≥ 48 mmol/mol (6.5%), were conducted using one-way ANOVA for normally distributed continuous data and Mann-Whitney *U* analyses for nonnormally distributed continuous data (diagnostic variables: HbA_1c_, total cholesterol, LDL, HDL, triglycerides, systolic and diastolic blood pressure, and BMI). Pearson chi-squared testing was used for comparisons of categorical data (ethnicity, age, gender, and the presence or absence of complications at recruitment and one year, as well as medication status). Binary logistic regression was performed to assess the association between demographic and diagnostic data (age at diagnosis, ethnicity, gender, diagnostic BMI, blood pressure, and lipid profile) and diagnostic HbA_1c_ category (< or ≥48 mmol/mol (6.5%)), accounting for multiple comparisons. Mann-Whitney *U* test was used to compare the mean rate of change in glycaemia between both groups.

The study was approved by King's College Hospital Research Ethics Committee (reference 08/H0808/1). All participants gave informed consent.

## 3. Results

**Figure 1:**
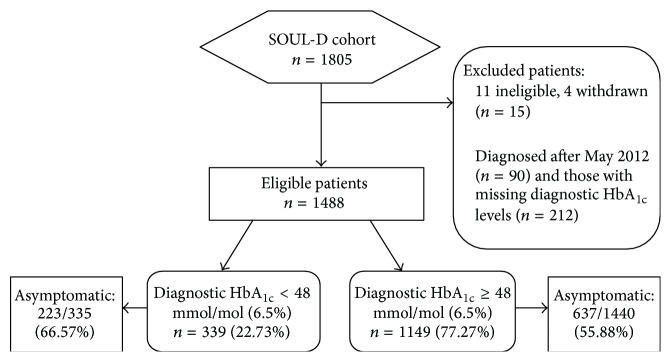
Flow chart for participants in the study.

Of 1805 participants recruited into SOUL-D, this analysis was restricted to 1715 participants diagnosed before May 2012 (Figure 1). Fifteen participants were excluded for failing to meet inclusion criteria or withdrawing themselves and 212 were excluded because HbA_1c_ was not documented at diagnosis. Of the remaining 1488, 55.11% were male (*n* = 820), mean age was 55.75 ± 11.02 years, and 50.13% (*n* = 746), 39.58% (*n* = 589), and 10.28% (*n* = 153) were of white, black, and South Asian/other ethnicity, respectively. Age and gender were not significantly different in those participants without HbA_1c_ at diagnosis and the population diagnosed after May 2012 (all *p* > 0.05).

Three hundred and thirty-nine participants (22.78%) had HbA_1c_ < 48 mmol/mol (6.5%) at the time of diagnosis by glucose criteria. This group was significantly older (58.73 ± 10.36 versus 54.87 ± 11.07; *p* < 0.0001) but had no significant difference in gender split (*p* = 0.883, 55.46% versus 55.00% male for the <48 mmol/mol and ≥48 mmol/mol (6.5%) resp.) groups versus the higher HbA_1c_ group Table 1. People of white ethnicity were overrepresented, while people of black and South Asian/other ethnicity were underrepresented (*p* < 0.0001). They had a significantly higher proportion of sensory neuropathy at recruitment but no significant differences in prevalence of retinopathy, microalbuminuria, MI, or stroke/CVA. In terms of nonglycemic cardiovascular risk at diagnosis, they had lower BMI and diastolic blood pressure, triglycerides, and total cholesterol (*p* < 0.01 for all) but no significant differences in systolic blood pressure, LDL-cholesterol, or HDL-cholesterol. All but 7 participants (4 with HbA_1c_ < 48 mmol/mol (6.5%)) had information on symptoms at presentation, with significantly more participants being asymptomatic at diagnosis in the low HbA_1c_ group (68.96% versus 55.41%, *p* = 0.0001). At recruitment, the participants with low HbA_1c_ at diagnosis group were significantly less likely to be receiving oral hypoglycemic agents (29.1% versus 61.9%, *p* < 0.0001) with a trend for fewer receiving insulin (1.78% versus 3.76%, *p* = 0.074). The group with low HbA_1c_ at diagnosis were more likely to be retired but there were no reported differences in proportion in full- or part-time employment or years of education attended (Table 2).

When demographic and diagnostic data (age at diagnosis, ethnicity, gender, diagnostic BMI, blood pressure, and lipid profile) were entered into a multiple binary logistic regression, only being black (*p* < 0.001), South Asian/other (*p* = 0.009), age at diagnosis (*p* = 0.02), and triglyceride levels at diagnosis (*p* = 0.001) were significant predictors of whether participants were diagnosed with HbA_1c_ < 48 mmol/mol (6.5%) or ≥48 mmol/mol (6.5%). The resulting model accounted for 7% of the variance and correctly identified 98.5% of cases.

### 3.1. Year One Analysis (Table 3)

Of the 1488 participants in the study, 21.51% (*n* = 320) were not available for follow-up at year 1: 6 had died; 262 were not contactable; and 52 had withdrawn from the study.

HbA_1c_ remained significantly lower in those participants with HbA_1c_ < 48 mmol/mol (6.5%) at diagnosis, *p* < 0.0001 (Table 3), and there was a significant difference in the change in HbA_1c_ from recruitment to year 1 between the two groups (*p* < 0.001). Participants with HbA_1c_ < 48 mmol/mol (6.5%) at diagnosis showed a slight increase in HbA_1c_ (median (IQR): 1.09 (−1.09–4.37) mmol/mol or 0.10 (−0.10–0.40)%), compared to a slight fall overall in the group with the high value at diagnosis (0.00 (−5.4645 to 4.3716) mmol/mol and 0.00 (−0.5000 to 0.400)%). BMI and diastolic blood pressure as well as smoking status were significantly lower and HDL cholesterol was significantly higher in the low HbA_1c_ at diagnosis group (*p* < 0.05 for all), with no significant differences in systolic blood pressure, LDL-cholesterol, total cholesterol, and triglycerides between groups. A higher proportion of those with low HbA_1c_ at diagnosis reported a new MI (3.38% versus 1.22%, *p* = 0.012). When the cumulative prevalence was compared between groups, that is, any MI events prior to year 1, the low HbA_1c_ group remained significantly more likely to have past MI (8.55% versus 5.14%, *p* = 0.019). Numbers were very small, but, while there were no differences in a post hoc analysis between those with and without a history of MI at recruitment, three of those reporting new MI at year one were of black ethnicity and one was of Asian/other ethnicity, with none being white. Significantly fewer participants in the lower HbA_1c_ group had developed new microalbuminuria (9.21% versus 15.65%, *p* = 0.012), and the cumulative prevalence of microalbuminuria prior to year 1 almost achieved significance (14.77% versus 20.02%, *p* = 0.054). Incidence of new retinopathy and stroke did not differ significantly between groups (all *p* > 0.1). A lower percentage of participants in the low HbA_1c_ at diagnosis group were receiving oral hypoglycemic agents (30.6% versus 71.9%, *p* < 0.0001) but the difference between groups in those receiving insulin at one year did not achieve statistical significance (2.16% versus 4.22%, *p* = 0.145).

### 3.2. Review of Diagnosis in Low Diagnostic HbA_1c_ Group (Table 4)

Information on diagnostic tests was obtained for 175 participants (51.62%) in the low HbA_1c_ at diagnosis group from 43 practices. These 175 participants were not significantly different from the remainder of the group in terms of demographical and biomedical data (*p* > 0.05) for all factors studied. There were 38.86%, 37.71%, 16.00%, and 7.42% participants diagnosed on basis of 2-hour plasma glucose, fasting plasma glucose, random plasma glucose, and HbA_1c_ measurements below the WHO recommendation (median (and IQR) for HbA_1c_ 46.45 (45.35–46.45) mmol/mol or 6.40 (6.30–6.40)%). Sixty-three (36.0%) asymptomatic patients, for whom data were available, had no repeat or subsequent alternative test documented. Eighty-nine (50.86%) participants, for whom data were available, fell outside prevailing WHO criteria for T2DM diagnosis.

## 4. Discussion

The aims of this study were to estimate the proportion of individuals in an urban multiethnic cohort that might not be diagnosed with T2DM if HbA_1c_ ≥ 48 mmol/mol is the sole diagnostic criterion and assess whether individuals diagnosed on alternative criteria and not meeting the new criterion differed in terms of demographics and biomedical outcomes from those with HbA_1c_ at diagnosis. In our cohort, almost a quarter of people previously diagnosed with T2DM would not be deemed diabetic, where HbA_1c_ was the sole diagnostic criterion. These people were older at diagnosis and more likely to be of white ethnicity. They were also more likely to have been asymptomatic at diagnosis. At recruitment, however, there was no difference in their complication status except for a higher prevalence of sensory neuropathy. One year later, although fewer were prescribed medical therapy for hyperglycemia, their HbA_1c_ remained lower but more had an MI in the year, although the group had lower BMI, diastolic blood pressure, and triglycerides throughout and lower prevalence and development of microalbuminuria. There were no significant differences in other micro- and macrovascular complications.

To our knowledge, this is the first study that has looked at the phenotypes associated with the different criteria for diagnosing T2DM. Other studies have found sole use of HbA_1c_ ≥ 48 mmol/mol (6.5%) to diagnose T2DM can alter the epidemiology of T2DM, reporting that diagnostic HbA_1c_ misses 62–65% of people (especially asymptomatic) identified on OGTT in screening programmes [15–17].

A high proportion of participants in both of our groups, significantly greater in those with low HbA_1c_ at diagnosis, denied osmotic symptoms at diagnosis. All diagnostic criteria require a second confirmatory biochemical test to establish the diagnosis in the absence of symptoms. Evidence of fulfilment of this requirement was absent from half the records investigated for this. Information not found in the patient or study records may have contributed to the diagnosis at the time. However, it is possible that in these participants another health event may have lowered physicians' threshold for diagnosing diabetes (reverse causality) [18]. The overrepresentation of retired people in the group may be a reflection of the slightly increased age, and/or a greater engagement with healthcare procedures, including screening. While healthcare is free at the point of delivery in the UK, cultural issues may influence uptake, although it should be noted that neither employment status nor educational attainment was different between the groups. Even with the potential for misdiagnosis of diabetes in some participants with low HbA_1c_ at diagnosis, the lack of difference in diabetes complications suggests that opportunities for secondary prevention may be missed by widespread use of HbA_1c_ alone to diagnose diabetes, for example, in screening programmes.

Nonglycemic cardiovascular risk factors such as BMI, lipid profile, and diastolic blood pressure were reduced in the low HbA_1c_ at diagnosis group and prevalence of smoking was not different. Nevertheless, although absolute numbers are low, there was a higher incidence of new MI at year one (with a possible trend towards a greater positive history of MI at recruitment). Possible contributors to this occurrence may include the slightly greater age of the subjects in this group, or their different ethnicity (see below), or reverse causality, with presence of a cardiovascular risk event increasing the chance of being screened for diabetes. Sensory neuropathy was also higher at recruitment in the group with the low diagnostic HbA_1c_. Sensory neuropathy is less specific to diabetes than retinopathy and nephropathy, although dysglycaemia has been implicated in the pathogenesis of otherwise idiopathic neuropathy [19–22]. The higher proportion of individuals on oral hypoglycemic agents in the higher HbA_1c_ group at recruitment and one year mirrors their average HbA_1c_ at those times. Given the lack of difference in complication status found between T2DM participants diagnosed with HbA_1c_ below or above the currently recommended guideline, our findings underline the importance of also applying non-HbA_1c_ tests.

Only ethnicity, age at diagnosis, and triglyceride levels predicted diagnosis with HbA_1c_ below 48 mmol/mol (6.5%) in multivariate analysis. Ethnicity may contribute to the differences in BMI, LDL cholesterol, and diastolic blood pressure noted at diagnosis and even to the higher development of MI in the first year after recruitment in the low diagnostic HbA_1c_ group. The SOUL-D participants of black ethnicity still show the traditional “cardioprotective” lipid profile, but they show higher blood pressure compared to white participants [11], and they were underrepresented in the low diagnostic HbA_1c_ group, although unexpectedly the incident MI in the study occurred in nonwhite participants. The proportion of people of black ethnicity recruited to SOUL-D precisely matches the proportion in registers of people with existing diabetes, suggesting that the SOUL-D study did pick up a representative sample of all those at risk for type 2 diagnosis and again arguing against healthcare access as a major contributor to the differences observed.

It is however likely that ethnicity itself impacts on HbA_1c_ at presentation of diabetes [23–25]. Higher HbA_1c_ in black people may not reflect higher plasma glucose concentrations and it has been suggested that plasma glucose might be more applicable than HbA_1c_ for diagnosing diabetes in black people [26]. However, two large population studies have shown that retinopathy prevalence is as high for any given HbA_1c_ and indeed may be higher than the reference category for a lower HbA_1c_ in black populations [27, 28]. This suggests HbA_1c_ is potentially stronger at predicting complications than plasma glucose and therefore a superior screening tool, as the main drive to diagnose asymptomatic diabetes is to engage in prevention of complications [27, 28]. Our finding of higher age at diagnosis in participants diagnosed with HbA_1c_ < 48 mmol/mol (6.5%), despite positive correlations between age and HbA_1c_ [29, 30], may relate to the younger age at diagnosis of diabetes in nonwhite populations [13].

Limitations include the exclusion of individuals with very advanced complications and those who were housebound or not fluent English language speakers. Exclusion of those with very advanced complications may have been greater in the higher HbA_1c_ group, although very advanced complications are not common in the newly diagnosed patients who were the target of this study. Nonfluent English language speakers might also be at higher risk for worse disease in that they may be less likely to access English language health services; however they formed only 7% potentially eligible patients [13]. The power of comparisons in biomedical data between diagnostic groups was weakened by a low number of micro- and macrovascular events in both groups. Positive aspects of the study were the large sample size and high representation of participants of black ethnicity, reflecting engagement with the specific multicultural population and their high risk for T2DM. The availability of diagnostic data, while not measured in a core laboratory, was analysed using similar assay methods and has allowed us to look at the characteristics of the subjects before intervention.

In conclusion, almost a quarter of SOUL-D study participants may not have been diagnosed with T2DM had HbA_1c_ been introduced as the sole diagnostic tool before their diagnosis. This may be of particular importance to asymptomatic individuals, where the purpose of diagnosis is to avoid future diabetes-related complications. This study provides evidence for the consideration of ethnicity when diagnosing T2DM using HbA_1c_: its use may miss people of white ethnicity who have previously been diagnosed on other criteria and may change the individuals identified with or without diabetes in screening programmes, perhaps particularly in older people and those of nonblack ethnicity. While long term follow-up of the cohorts reported here will be confirmatory, our present findings so suggest that while HbA_1c_ of 48 mmol/mol (6.5%) or greater as the diagnostic marker for T2DM is useful when positive, a negative result cannot be taken as conclusive evidence to exclude the diagnosis of clinically important diabetes.

## Figures and Tables

**Table 1:** Comparisons between participants diagnosed with HbA_1c_ < 48 mmol/mol and with HbA_1c_ ≥ 48 mmol/mol at diagnosis and recruitment.

	*n* with data	HbA_1c_ < 48 mmol/mol (6.5%)	HbA_1c_ ≥ 48 mmol/mol (6.5%)	*p* value
Demographical data				
Age	1488	58.73 (±10.36)	54.87 (±11.07)	<0.0001
Gender (% male)	1488	188 (55.46)	632 (55.00)	0.8829
Ethnicity (%)	1488			
White		218 (64.31)	528 (45.95)	<0.0001
Black		98 (28.91)	491 (42.73)	
Asian		23 (6.78)	130 (11.31)	

Median (IQR) HbA_1c_ at diagnosis				
HbA_1c_ (mmol/mol)	1488	44.26 (42.08–45.35),	58.47 (51.91–82.52)	<0.0010
HbA_1c_ (%)		6.20 (6.00–6.30)	7.50 (6.90–9.70)	

Cardiovascular risk factors (SD) at diagnosis				
LDL-C (mmol/L)	1211	2.86 (±0.98)	3.67 (±21.83)	0.5313
HDL-C (mmol/L)	1251	1.28 (±0.67)	1.24 (±0.44)	0.1750
Total cholesterol (mmol/L)	1357	4.70 (±1.32)	4.90 (±1.50)	0.0053
Triglycerides (mmol/L)	1273	1.40 (±0.85)	1.59 (±1.29)	0.0001
BMI (kg/m^2^)	1380	30.77 (±7.74)	31.60 (8.40)	0.0035
BPS (mmHg)	1409	133.96 (±16.84)	134.65 (±16.28)	0.5087
BPD (mmHg)	1409	80.00 (±11.00)	82.00 (±12.00)	<0.0001

Complication status, HbA_1c_ medication status at recruitment				
Retinopathy (%)	1335	30 (9.43)	80 (7.86)	0.3749
Microalbuminuria (%)	1237	37 (12.76)	158 (16.68)	0.1279
Sensory neuropathy (%)	1368	32 (10.16)	70 (6.65)	0.0393
MI (%)	1482	24 (7.08)	55 (4.81)	0.1026
Stroke (%)	1479	8 (2.37)	40 (3.50)	0.3042
HbA_1c_ (mmol/mol)	1395	42.08 (38.80–44.26)	50.82 (45.35–60.65)	<0.0001
HbA_1c_ (%)		6.00 (5.70–6.20)	6.80 (6.30–7.40)	
Receiving insulin (%)	1481	6 (1.78)	43 (3.76)	0.0743
Receiving oral diabetes agents (%)	1470	97 (29.1)	704 (61.9)	<0.0001

**Table 2:** Employment and educational status.

	HbA_1c_ ≥ 48 mmol/mol (6.5%)	HbA_1c_ < 48 mmol/mol (6.5%)
In full-time employment (%)	430 (37.42)	119 (35.10)
In part-time employment (%)	123 (10.70)	36 (10.62)
On sick leave (%)	23 (2.00)	9 (2.65)
Unemployed (%)	189 (16.45)	34 (10.03)
Medically retired (%)	51 (4.44)	18 (5.31)
Housewife/househusband (%)	43 (3.74)	6 (1.77)
Retired (%)	289 (25.15)	117 (34.51)^∗^

Education status		
Years of education (mean ± SD)	13.19 ± 2.96	13.50 ± 3.15

^∗^ *p* = 0.0009, all other comparisons not significant.

**Table 3:** Prospective comparisons between participants diagnosed with HbA_1c_ < 48 mmol/mol and those diagnosed with HbA_1c_ ≥ 48 mmol/mol at year 1.

	*n* with data	HbA_1c_ < 48 mmol/mol (6.5%)	HbA_1c_ ≥ 48 mmol/mol (6.5%)	*p* value
Median (IQR) HbA_1c_ at year 1				
HbA_1c_ (mmol/mol)	1251	43.17 (40.98–47.54),	49.72 (45.35–57.37)	<0.0001
HbA_1c_ (%)		6.10 (5.90–6.50)	6.70 (6.30–7.40),	

New complication status at year 1				
Retinopathy (%)	1220	32 (10.88)	126 (13.61)	0.2257
Microalbuminuria (%)	1025	22 (9.21)	123 (15.65)	0.0123
MI (%)	1278	10 (3.38)	12 (1.22)	0.0124
Stroke (%)	1272	4 (1.4)	7 (0.72)	0.2951

Cumulative microalbuminuria and MI status at year 1				
Microalbuminuria (%)	1218	39 (14.77)	191 (20.02)	0.0540
MI (%)	1487	29 (8.55)	59 (5.14)	0.0190

Medication status at year 1				
Receiving oral agents (%)	1296	92 (30.6)	715 (71.9)	<0.0001
Receiving insulin (%)	1294	8 (2.67)	37 (3.72)	0.3817

Cardiovascular risk factor status (SD) at year 1				
BMI (kg/m^2^)	1308	31.28 (±6.44)	32.14 (±6.35)	0.0400
BPD (mmHg)	1282	80.21 (±10.76)	82.22 (±10.91)	0.0057
BPS (mmHg)	1284	135.42 (±18.28)	134.57 (±17.23)	0.4614
LDL-C (mmol/L)	1164	2.49 (±0.80)	2.51 (±0.84)	0.7485
HDL-C (mmol/L)	1200	1.32 (±0.48)	1.25 (±0.34)	0.0081
Total cholesterol (mmol/L)	1234	4.40 (±1.30)	4.40 (±1.20)	0.0942
Triglycerides (mmol/L)	1164	2.49 (±0.80)	2.51 (±0.84)	0.5454
Smoking status (%)	1149	190 (21.62)	41 (15.19)	0.0211

**Table 4:** Basis for diagnosis in patients with HbA_1c_ < 48 mmol/mol.

	Basis of diagnosis	Asymptomatic and repeat test not performed	Incorrectly diagnosed according to WHO
Fasting plasma glucose	66 (37.71%)	18 (27.27%)	26 (39.39%)
OGTT	68 (38.86%)	21 (30.88%)	34 (50.00%)
Random plasma glucose	28 (16.00%)	12 (42.86%)	15 (53.57%)
HbA_1c_	13 (7.42%)	5 (38.46%)	13 (100%)

Total (%)	175	63 (36.00%)	89 (50.86%)

## Abbreviations

CVA: Cerebrovascular incident
HbA_1c_: Glycated haemoglobin
MI: Myocardial infarction
T2DM: Type 2 diabetes mellitus
ACR: Albumin creatinine ratio
BMI: Body mass index
OGTT: Oral glucose tolerance test.
